# Probiotic OMNi-BiOTiC^®^ 10 AAD Reduces Cyclophosphamide-Induced Inflammation and Adipose Tissue Wasting in Mice

**DOI:** 10.3390/nu15163655

**Published:** 2023-08-20

**Authors:** Beate Obermüller, Georg Singer, Bernhard Kienesberger, Barbara Mittl, Vanessa Stadlbauer, Angela Horvath, Wolfram Miekisch, Patricia Fuchs, Martina Schweiger, Laura Pajed, Holger Till, Christoph Castellani

**Affiliations:** 1 Department of Paediatric and Adolescent Surgery, Medical University of Graz, 8036 Graz, Austria; beate.obermueller@medunigraz.at (B.O.); bernhard.kienesberger@medunigraz.at (B.K.); barbara.mittl@medunigraz.at (B.M.); holger.till@medunigraz.at (H.T.); christoph.castellani@medunigraz.at (C.C.); 2 Department of Paediatric Surgery, Clinical Center of Klagenfurt, 9020 Klagenfurt, Austria; 3 Division of Gastroenterology and Hepatology, Department of Internal Medicine, Medical University of Graz, 8036 Graz, Austria; vanessa.stadlbauer@medunigraz.at; 4 Center of Biomarker Research (CBmed), 8010 Graz, Austria; angela.horvath@medunigraz.at; 5 Department of Anesthesiology and Intensive Care & Pain Therapy, Rostock University Medical Center, 18057 Rostock, Germany; wolfram.miekisch@uni-rostock.de (W.M.); patricia.fuchs@uni-rostock.de (P.F.); 6 Institute of Molecular Biosciences, BioTechMed-Graz, BioHealth-Graz, University of Graz, 8010 Graz, Austria; tina.schweiger@uni-graz.at (M.S.); laura.pajed@uni-graz.at (L.P.); 7 Department of Anesthesiology and Intensive Care Medicine, Weiz District Hospital, 8160 Weiz, Austria

**Keywords:** chemotherapy, inflammation, microbiome, gut permeability, weight loss, volatile organic compound, histomorphometry

## Abstract

Cancer therapy is often associated with severe side effects such as drug induced weight loss, also known as chemotherapy-induced cachexia. The aim of this study was to investigate the effects of a multispecies probiotic (OMNi-BiOTiC^®^ 10 AAD) in a chemotherapy mouse model. A total of 24 male BALB/c mice were gavage-fed with the probiotic formulation or water, once a day for 3 weeks. In the third week, the mice received intraperitoneal cyclophosphamide. At euthanasia, the organs were dissected, and serum was sampled for cytokine analysis. Tight junction components, myosin light chain kinase, mucins, and apoptosis markers were detected in the ileum and colon using histological analyses and qRT-PCR. Lipolysis was analyzed by enzymatic activity assay, Western blotting analyses, and qRT-PCR in WAT. The fecal microbiome was measured with 16S-rRNA gene sequencing from stool samples, and fecal volatile organic compounds analysis was performed using gas chromatography/mass spectrometry. The probiotic-fed mice exhibited significantly less body weight loss and adipose tissue wasting associated with a reduced CGI58 mediated lipolysis. They showed significantly fewer pro-inflammatory cytokines and lower gut permeability compared to animals fed without the probiotic. The colons of the probiotic-fed animals showed lower inflammation scores and less goblet cell loss. qRT-PCR revealed no differences in regards to tight junction components, mucins, or apoptosis markers. No differences in microbiome alpha diversity, but differences in beta diversity, were observed between the treatment groups. Taxonomic analysis showed that the probiotic group had a lower relative abundance of *Odoribacter* and *Ruminococcus-UCG014* and a higher abundance of *Desulfovibrio*. VOC analysis yielded no significant differences. The results of this study indicate that oral administration of the multispecies probiotic OMNi-BiOTiC^®^ 10 AAD could mitigate cyclophosphamide-induced chemotherapy side effects.

## 1. Introduction

Chemotherapy is an important cornerstone of the modern management of malignant tumors. However, adverse effects of chemotherapy, such as intestinal mucositis or chemotherapy induced diarrhea, have been reported in 50–80% of patients [1,2]. In addition to significant effects on the patients’ quality of life, these side effects may warrant dose reduction or even discontinuation of chemotherapeutical agents, resulting in increased mortality [3,4]. Intestinal mucositis (IM) clinically presents with nausea, vomiting, bloating, diarrhea or constipation, abdominal pain, or body weight loss [5,6]. A disturbance of the integrity of the intestinal barrier due to epithelial cell loss, in combination with inflammation of the bowel wall, seems to be the main reason for the development of IM [6,7,8]. The impaired epithelial lining may also be predisposed to bacterial translocation, with subsequent severe systemic inflammation [9].

Examinations of the intestinal microbiome in chemotherapy models have revealed a possible role of intestinal bacteria in both the development and severity of IM [10,11]. In general, chemotherapy causes a decrease in *Lactobacillus*, *Bifidobacterium*, and other protective bacteria and an increase in potential pathogens [12]. An increase in *Enterococcus*, *Enterobacter*, *Escherichia*, and *Pseudomonas* has been described in a murine chemotherapy model [13]. The alterations of the microbiome observed in this study were associated with increased intestinal permeability and the reduced expression of tight junction proteins [13]. These and other findings suggest that intestinal dysbiosis plays a significant role in IM, thus triggering the search for possibilities to improve chemotherapy by supporting the intestinal microbiome. 

In this regard, several probiotics were shown to reduce chemo- and radiotherapy-induced diarrhea and mucositis (reviewed in [14]). For instance, *Lactobacillus* and *Lachnospiraceae* were shown to decrease the side effects of cyclophosphamide (CTX). The restoration of eubiotic conditions by lowering the pH and the direct inhibition of pathogens by the secretion of antimicrobial substances have been discussed as possible probiotic effects [15,16]. Furthermore, *Lactobacilli* and *Bifidobacteria* help to restore the intestinal barrier by improving the expression of tight junctions and mucus secretion from goblet cells [17,18,19].

In addition to improving chemotherapy side effects, certain probiotics promote cancer cell apoptosis (predominantly *Lactobacillus* spp., as reviewed in [14]). Furthermore, gut microbiota (as *Lactobacillus* spp. or *Enterococcus hirae*) may also directly enhance the effectiveness of chemotherapeutical agents [14]. Consequently, studies have demonstrated that an intact microbiome is essential for effective chemotherapy [20]. On the cellular level, tumor suppressor gene activation, cell cycle maintenance, and the inactivation of proinflammatory cytokines and oncogenes have been reported as possible mechanisms for the positive effects of probiotics [21,22]. Overall, the combined action of probiotics and anti-cancer drugs showed increased anti-tumor efficiency (reviewed in [14]). 

In this study, we aimed to examine the effect of a combination of nine probiotic strains (in OMNi-BiOTiC^®^ 10 AAD) on systemic inflammation, body weight loss, and adipose tissue wasting, as well as the fecal microbiome and volatile organic compounds (VOCs) during CTX chemotherapy in a murine model.

## 2. Materials and Methods

Following approval of the veterinary board (2020-0.475.510), 24 male BALB/c mice were obtained from the Center for Biomedical Research of the Medical University of Vienna, Austria, as one batch of littermates for microbiome testing at an age of 7 weeks. After delivery and an acclimatization period of 2 weeks, the mice were divided into two equal groups (*n* = 12 each), with comparable body weight distribution. The mice were housed in groups of three animals in individually ventilated cages under specific pathogen free conditions, with a 12 h light-dark cycle and free access to chow and water at all times. OMNi-BiOTiC^®^ 10 AAD was kindly provided by the Institute AllergoSan (Graz, Austria). OMNi-BiOTiC^®^ 10 AAD is a commercially available multispecies probiotic formulation containing the strains *Lactobacillus acidophilus W55* and *W37*, *Lactobacillus paracasei W20*, *Lactobacillus rhamnosus W71*, *Enterococcus faecium W54*, *Lactobacillus salivarius W24*, *Lactobacillus plantarum W62*, *Bifidobacterium bifidum W23*, and *Bifidobacterium lactis W51*. After acclimatization, the mice received a daily gavage of either 12 × 10^8^ CFU OMNi-BiOTiC^®^ 10 AAD dissolved in 400 µL sterile water (10 AAD group) or 400 µL of sterile water only (AQUA group). Gavage was continued daily until euthanasia. 

After 3 weeks, CTX chemotherapy was initiated in both groups. CTX was prepared as a 10 mg/mL solution in physiologic saline and administered by intraperitoneal injection. Dosages were as follows: 100 mg/kg body weight on days 1–3, a break for 1 day, followed by 50 mg/kg on days 5 and 6. On day 7, stool samples were harvested to standard Eppendorf vials and stored at −80 °C for subsequent microbiome analysis. The mice were then gavage-fed with 500 mg/kg fluorescein-isothiocyanate dextrane 4kD (FITC-Dextran 4SD, Sigma-Aldrich Handels GesmbH, Vienna, Austria) dissolved in PBS buffer at a concentration of 50 mg/mL.

A second stool sample was obtained, weighed for normalization, and stored in standard glass vials (Gerstel GmbH, Muelheim an der Ruhr, Germany) at 6 °C. Room air samples were collected at the same time points to correct for possible contamination. These fecal and air samples were sent by overnight express to the partner lab in Rostock, Germany, for subsequent volatile organic compound (VOC) analysis. Euthanasia was conducted exactly 16 h after FITC administration. The mice were then put under inhalation anesthesia using 5% Isoflurane^®^ (Forane^®^, Baxter Healthcare GmbH, Vienna, Austria). Blood was drawn to standard serum vials and allowed to clot for 30 min. Then, blood samples were centrifuged at 4 °C with 10,000 rpm for 10 min. The supernatant serum was harvested and aliquoted to standard Eppendorf vials. The mice were sacrificed by cranio-cervical dislocation.

White adipose tissue (WAT) was sampled from the intestinal, inguinal, and perirenal regions, as previously described [23]. The following organs were obtained for further analysis: liver, spleen, kidney, triceps surae muscle, 3 cm of the ileum (beginning 1 cm orally of the ileocecal valve), and 3 cm of the colon (beginning 1 cm distally of the ileocecal valve). All specimens were labeled with the mouse number, blinding examiners for the experimental groups.

Throughout the experiment, the body weight was recorded twice a week in the first 3 weeks and daily during CTX therapy. Food pellets were weighed at the same time points and used to estimate the caloric intake per cage.

### 2.1. Inflammatory Response

A commercially available Procartaplex^®^ magnetic bead assay (Procartaplex, Thermo Fisher Life Tech Austria, Vienna, Austria) was configured to detect interleukins (IL) 1α, IL-1β, IL-6, IL-10, IL-15, and IL-17; monocyte chemoattractant protein (MCP) 1; macrophage inflammatory proteins (MIP) 1α, MIP-1β, and MIP-2; granulocyte colony stimulating factor (G-CSF); granulocyte macrophage colony stimulating factor (GM-CSF); macrophage colony stimulating factor (M-CSF); TNF-α, vascular endothelial growth factor (VEGF); and transforming growth factors (TGF) β1 and β2 in the serum samples. Myeloperoxidase (MPO, AB275109, Abcam, Cambridge, UK), lipoprotein binding protein (LBP, ABIN6974448, antikoerper-online.de, Aachen, Germany), and neutrophil elastase (NE, AB252356, Abcam, Cambridge, UK) were determined using commercially available ELISA kits. All tests were performed as described in the manufacturer’s instructions.

### 2.2. Gut Permeability Assay and Bowel Wall Integrity

For FITC analysis, the serum aliquots were protected from light and stored at 6 °C until measurement. FITC-Dextrane serum levels were determined within 8 h after photometrical harvesting (FLUOstar Omega, BMG LABTECH, Ortenberg, Germany) at an extinction rate of 485 and 535 nm. Standard curves were obtained, according to the manufacturer´s protocol.

For the examination of the bowel wall integrity, the total RNA from the frozen mouse bowel segments (ileum and colon) was isolated using the Qiagen miRNeasy Micro Kit (Qiagen, Hilden, Germany) by DNAse treatment (Qiagen, Hilden, Germany), according to manufacturer’s instructions. The RNA yield was quantified using a NanoDrop 2000 c spectrophotometer. For reverse transcription, 1 µg of total RNA was used in the High-Capacity cDNA Reverse Transcription Kit (ThermoFisher Scientific, Waltham, MA, USA), according to the manufacturer’s instructions. The cDNA product was used as a template for quantitative RT-PCR reactions in a BioRad CFX 384 real-time PCR detection system with the following assays: β-actin (Actb), hydroxymethylbilane (Hmbs), tight junction protein 1 (Tjp1), occludin-1 (Ocln1), claudin 4 (Cldn4), claudin 2 (Cldn2), mucin 2 (MUC2), mucin 3 (MUC3), myosin light chain kinase (MLCK), B-cell lymphoma 2 (Bcl2), Bcl-2-associated X protein (Bax), Bcl-2 associated agonist of cell death (Bad), caspase 3 (Casp3), lamin B1 (Lmnb), and Bcl-2 antagonist/killer 1 (Bak1). All primers were ordered from Eurofins Genomics (Eurofins Genomics GmbH, Ebersberg, Germany). Detailed information on primer sequences can be retrieved from the Supplementary Material (Supplementary Table S1).

Briefly, in 10 µL reactions, 4 µL cDNA were used in triplicate in a PCR reaction with 5 µL TaqMan Genexpression MasterMix (ThermoFisher Scientific, Waltham, MA, USA), 0.5 µL assay, and 0.5 µL dH_2_O. The cycling conditions consisted of an initial UDG incubation at 50 °C for 2 min, enzyme activation at 95 °C for 10 s, followed by 40 cycles of denaturation at 95 °C for 15 s and annealing and extension at 60 °C for 1 min. Β-actin and Hmbs genes were used for normalization. 

### 2.3. Lipolysis Markers

qRT-PCR analysis: The following genes were analyzed: adipocyte triglyceride lipase (ATGL), hormone sensitive lipase (HSL), perilipin 1 (PLIN1), and comparative gene identification 58 (CGI58) (Supplementary Table S1). Hmbs and Actb served as controls for normalization. All primers were ordered from Eurofins Genomics (Eurofins Genomics GmbH, Ebersberg, Germany).

Western blotting analysis: The tissues were disrupted in ice-cold solution A (0.25 M sucrose, 1 mM EDTA, pH 7.0) supplemented with 1% NP40, protease inhibitor (20 μg/mL leupeptin, 2 μg/mL antipain, and 1 μg/mL pepstatin), and phosphatase inhibitor (phosstop, Sigma Aldrich, St. Louis, MO, USA). The homogenates were centrifuged for 30 min at 4 °C and 16,000× *g*, the lipid layer was removed, and the protein concentration of the infranatant was determined using Protein Assay Dye (Bio-Rad Laboratories, Hercules, CA, USA). A total of 10 µg protein was subjected to SDS-PAGE. After SDS-PAGE, the proteins were blotted onto a methanol-activated polyvinylidenfluorid (PVDF) membrane (Carl Roth GmbH, Karlsruhe, Germany) for 100 min at 200 mA. Unspecific binding sites were blocked using 5% milk powder or 5% BSA (Carl Roth GmbH, Karlsruhe, Germany) in 1× TST, followed by incubation with a primary and an HRP-conjugated secondary antibody (vinculin: Sigma V9131 1:20,000 4 °C; pHSL(Ser563): cell signaling #4139 1:2000 at room temperature (RT); tHSL: cell signaling #4107 1:7000 at RT. CGI58: abnova #H00051099-M01 at 4 °C; ATGL: cell signaling #2138 1:5000 at RT). HRP-conjugated antibodies were detected by chemiluminescence using Clarity Western Enhanced chemiluminescence (ECL) substrate (Bio-Rad Laboratories) and ChemiDoc Touch Imaging System (Bio-Rad Laboratories). Signal intensities were determined by densitometric analyses using Image Lab software (Bio-Rad Laboratories). Vinculin was used as housekeeping protein.

The triglyceride hydrolase activity assay was performed as described in the research of [24], with some modifications: adipose tissue powder was homogenized in ice-cold solution A (0.25 M sucrose, 1 mM DTT, 1 mM EDTA, pH 7), supplemented with protease inhibitor (20 μg/mL leupeptin, 2 μg/mL antipain, and 1 μg/mL pepstatin), using Ultra-Turrax Homogenizer. The homogenate was centrifuged for 30 min at 20,000× *g* and 4 °C. The supernatant, without fat cakes, was collected and centrifuged as described above, and infranatant was used for measuring TG hydrolase activity. The triglyceride substrate contained 1.67 mM triolein (TO), 10 µCi/mL [3H]-TO as a tracer, and 188 µM PC/PI (3/1, M/M). The lipids were fully evaporated and emulsified by sonication in 100 mM potassium phosphate buffer (pH 7.0). Thereafter, fatty acid-free BSA was added to achieve a final concentration of 5%. For the assay, 25 µL lysate (20 µg protein) and 25 µL substrate were incubated for 1 h at 37 °C. The enzymatic reaction was terminated by the addition of 650 µL STOP solution (methanol/chloroform/*n*-heptane; 10/9/7, *v*/*v*/*v*), followed by 200 µL potassium-carbonate (pH 10.5). The mixture was vortexed twice for 10 s and centrifuged at 2000× *g* for 10 min, and 200 µL of the upper phase was used to determine the radioactivity by liquid scintillation counting.

### 2.4. Bowel Wall Inflammation

For the visual determination of bowel wall inflammation, the ileum and colon sections underwent standard histological processing and hematoxylin and eosin staining. The morphology and inflammation in the bowel wall of the ileum were classified using the Marsh–Oberhuber Score [25]. Colonic inflammation and histomorphology were scored according to the methods of Erben et al. [26].

### 2.5. Fecal Microbiome Analysis

For total DNA isolation, fecal samples were isolated with the Magna Pure LC DNA III Isolation Kit (Bacteria, Fungi) (Roche, Mannheim, Germany), according to published protocols [27]. Briefly, one stool pellet was mixed with 500 µL PBS and 250 µL bacterial lysis buffer. The samples were homogenized and bead beaten in Magna Lyser Green bead Tubes (Roche, Mannheim, Germany) in a Magna Lyser instrument (Roche, Mannheim, Germany) twice at 6500 rpm for 30 s. This process was followed by enzymatic lysis with 25 µL lysozyme (100 ng/mL, 37 °C for 30 min) and 43.4 µL proteinase K (20 mg/mL, 65 °C for 1 h); the samples were heat inactivated at 95 °C for 10 min, and the total DNA was purified in a MagnaPure LC instrument, according to the manufacturer´s instructions. The total DNA was eluted in 100 µL elution buffer and stored at −20 °C until analysis. For 16S PCR, 2 µL of total DNA was used as a template in a 25 µL PCR reaction using the FastStart™ High Fidelity PCR-System (Sigma, Darmstadt, Germany), according to the manufacturer’s instructions, and the target specific primers were 515F (5′-GTGYCAGCMGCCGCGGTAA-3′) and 806R (5′-GGACTACNVGGGTWTCTAAT-3′) for 30 cycles, in triplicate. The triplicates were pooled, normalized, indexed and purified according to published protocols [27]. The final pool was sequenced on an Illumina MiSeq desktop sequencer at 9 pM and v 3 600 cycles chemistry. FASTQ raw files were used for data analysis. 

A total of 2,479,083 MiSeq paired-end FASTQ reads were used for further analysis. The DADA2 pipeline for modeling and correcting Illumina-sequenced amplicon errors for quality-filtering [28] was used, with standard settings for denoising, dereplicating, merging, and check for chimeras, as implemented in the QIIME2 2018.4 microbiome bioinformatics platform [29]. QIIME2 was integrated in an own non-public instance of Galaxy (MedBionode https://galaxy.medunigraz.at) [30]. Taxonomic assignment of the DADA2 representative sequences was obtained using the QIIME2 sklearn-based classifier for the SILVA rRNA database release 132 at 99% identity [31]. Galaxy based tools were used to calculate alpha (Inversed Simpson, Chao1 and Shannon) and beta (weighted and unweighted UniFrac) diversity markers. Relative abundances at the different levels were retrieved and used for group comparison. ANCOM, LEfSE, and Random Forest were used to locate discriminating biomarkers.

### 2.6. Fecal Volatile Organic Compounds

All fecal samples were analyzed within 48 h. VOC samples were drawn from the headspace of the fecal samples, as previously reported [32,33,34]. VOCs were pre-concentrated with a commercially available solid phase micro extraction (SPME) fiber (carboxen/polymethylsiloxane, Supelco, Bellefonte, PA, USA), which was thermally desorbed and analyzed by gas chromatography/mass spectrometry (GC/MS). An Agilent 7890 A gas chromatograph (GC) coupled to an Agilent 5975 C inert XL mass selective detector (MSD) was used to separate and identify the VOCs desorbed from the SPME device. The detected marker substances were tentatively identified from a mass spectral library (National Institute of Standards and Technology 2005; NIST 2005, Gatesburg, PA, USA), as well as using retention time matching. The results were corrected for the stool weight. Room air was sampled in parallel to the fecal samples. In case the mean of the room air samples exceeded 30% of the mean of the headspace samples, a possible contamination was recorded, and the substance was excluded from further analysis. The responses of a selected m/q ratio at a defined retention time for each substance were recorded, integrated, and used for group comparison.

### 2.7. Statistical Analysis

The data were managed with Microsoft Excel 2019^®^ (Microsoft Corporation, Redmond, WA, USA) spreadsheets. Statistical analysis was conducted with SPSS 26.0^®^ (IBM Corporation, Armonk, NY, USA). Due to the small sample size, normal distribution could not be assumed. Hence, a non-parametric Mann–Whitney U test was used for group comparison, and a Spearman test was used for correlation analysis. A result of *p* < 0.05 was considered statistically significant.

A graphical workup was obtained with Prism 9^®^ (GraphPad, San Diego, CA, USA). R studio^®^ (version 1.4.1106) was used for correlation analysis with a Spearman–Rho test and the corrplot package (version 0.92) [35]. Additionally, correlation paths were visualized with corrr (version 0.4.3) [36], tidyverse (version) [37], and farver (version 2.1.0) [38] packages.

## 3. Results

**Figure 1:**
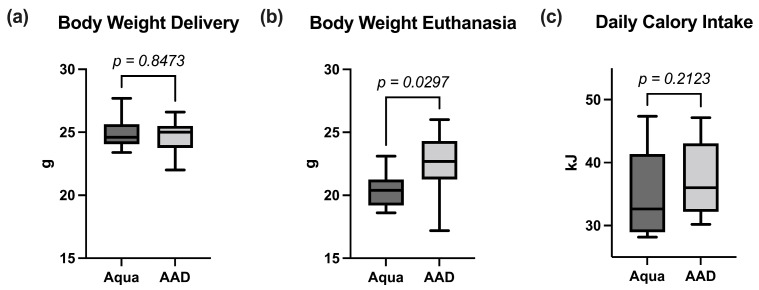
Nine-week-old mice received a daily gavage of either 10 AAD (AAD) or water (Aqua) for 4 weeks. (**a**) At delivery, mice were weighed (body weight delivery) and treated with CTX for one week. (**b**) Body weights at euthanasia. (**c**) Daily calorie intake during CTX therapy was determined by weighing the amount of food in the hoppers to estimate the mean daily calorie intake of the two groups. Data are shown as median ± interquartile range (*n* = 9 per group).

**Figure 2:**
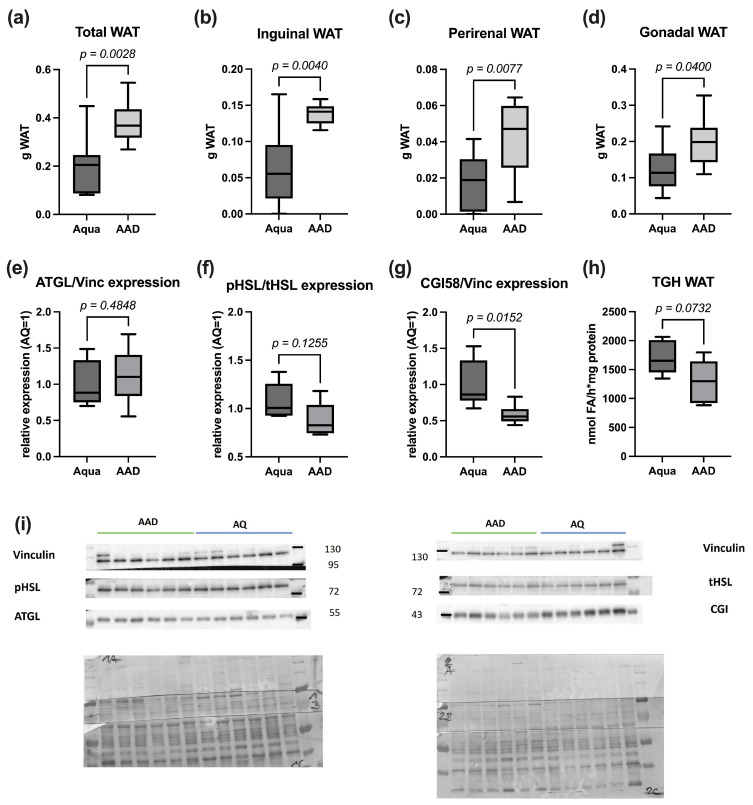
Tissue weights of total (**a**), perirenal (**b**), gonadal (**c**), and inguinal (**d**) WAT of 10 AAD or water (Aqua) treated animals after 1 week of CTX treatment. Western blotting analysis of ATGL (**e**); HSL, pHSL (Ser563) (**f**); and CGI-58 (**g**) in WAT Triglyceride hydrolase (TGH); activity of WAT (**h**); Western blot (**i**). Data are shown as median ± interquartile range (*n* = 9 per group (**a**–**d**); *n* = 6 per group (**e**–**i**)).

In the control and the 10 AAD treated group, three animals died during CTX therapy, leaving nine animals in each group for final analysis. While the initial body weight distribution was not significantly different between the groups, the animals in the 10 AAD group lost significantly less weight during chemotherapy (Figure 1a,b). The daily calorie intake during the CTX therapy, however, did not differ significantly between the groups (Figure 1c). While other organs did not differ in their weight, 10 AAD animals exhibited significantly more WAT than those of the control group (Figure 2a–d).

### 3.1. Inflammatory Response

**Figure 3:**
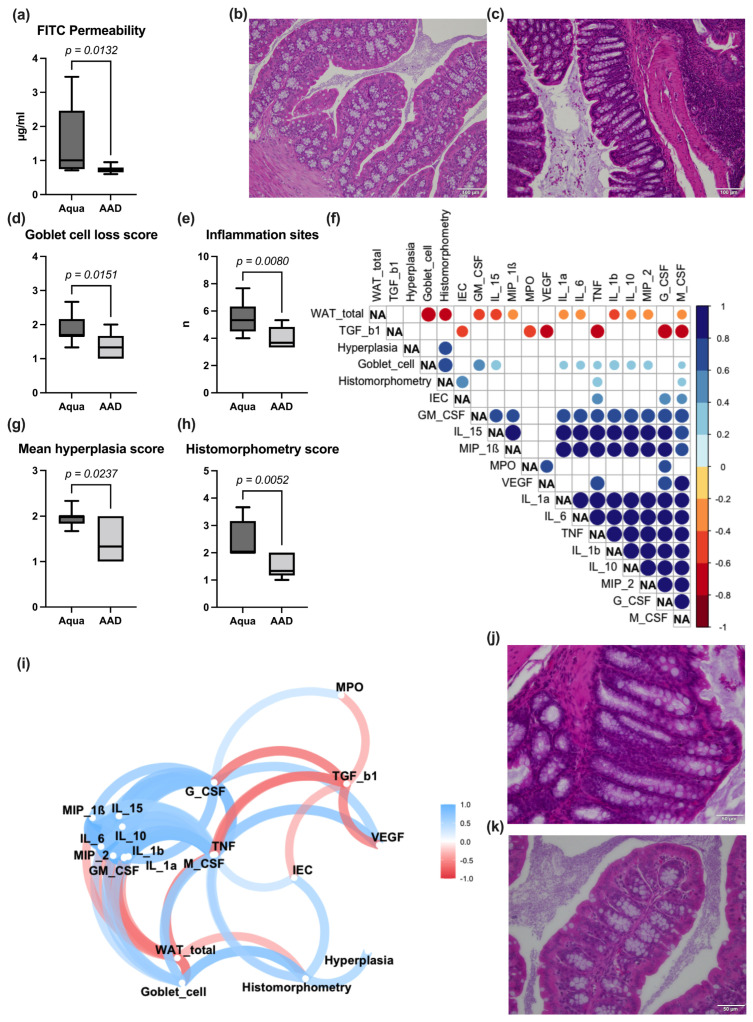
After 3 weeks of 10 AAD/Aqua treatment, the mice were treated with CTX for another week. Thereafter, the mice were sacrificed. FITC permeability (**a**); histological slides of ileum samples of the Aqua (**b**) and 10 AAD (**c**) group (H&E staining, 400× magnification). Histomorphometry markers for inflammation (**d**,**e**,**g**,**h**) in the colon. Correlation analysis (**f**) (only significant correlations are displayed). Correlation paths (**i**) and colon histology (H&E staining, 400× magnification) of Aqua (**j**) and 10 AAD (**k**). Data are shown as median ± interquartile range (*n* = 9 per group).

The 10 AAD group exhibited significantly lower serum levels of most pro-inflammatory cytokines (IL-1α, IL-1β, IL-6, IL-15, MIP-1β, MIP-2, TNF-α, G-CSF, GM-CSF, M-CSF, MPO, and VEGF), while the anti-inflammatory cytokine TGF-β1 was significantly higher in the 10 AAD group (Table 1). The WAT mass significantly correlated with selected pro-inflammatory cytokines (Figure 3f). 

### 3.2. Lipolysis Markers

Adipose tissue loss is often associated with an increased degradation of lipid stores by lipases. Despite significantly more WAT (Figure 2) in the 10 AAD animals compared to in the control mice, there were no differences in the expression of genes encoding for Atgl, Hsl, Cgi58, and Plin1. Due to a limited amount of WAT per animal, six samples per group remained for protein analysis. The protein levels of ATGL and HSL, the two main lipases of WAT, were not significantly different between the groups (Figure 2e,f). However, 10 AAD animals exhibited a trend towards the reduced phosphorylation of Ser563, indicating the decreased hydrolytic activity of HSL (Figure 2f). Additionally, the WAT of 10 AAD animals showed significantly less CGI-58 protein expression, indicating lower ATGL-activation (Figure 2g). These expression data were indicative of reduced triglyceride hydrolase activity in the WAT of 10 AAD animals, compared to the data for the controls (Figure 2h).

### 3.3. Gut Permeability Assay, Bowel Wall Integrity, and Inflammation

After CTX treatment, mice in the 10 AAD group exhibited a significantly decreased permeability for FITC (Figure 3a), but not for LPB (Table 1) when compared to the control group. While there was no significant difference in the morphology of the ileum (villus and crypt height, Marsh–Oberhuber Score), we encountered significant alterations of the colonic architecture in the Aqua group (higher number of inflammation sites, increased goblet cell loss, increased bowel wall inflammation (as measured in the histomorphometry score; Figure 3d,e,h), and higher intestinal hyperplasia (Figure 3g). Correlation analysis revealed that the serum levels of pro-inflammatory cytokines and the total WAT of the animals significantly correlated with each other (Figure 3f).

### 3.4. Fecal Microbiome Analysis

**Figure 4:**
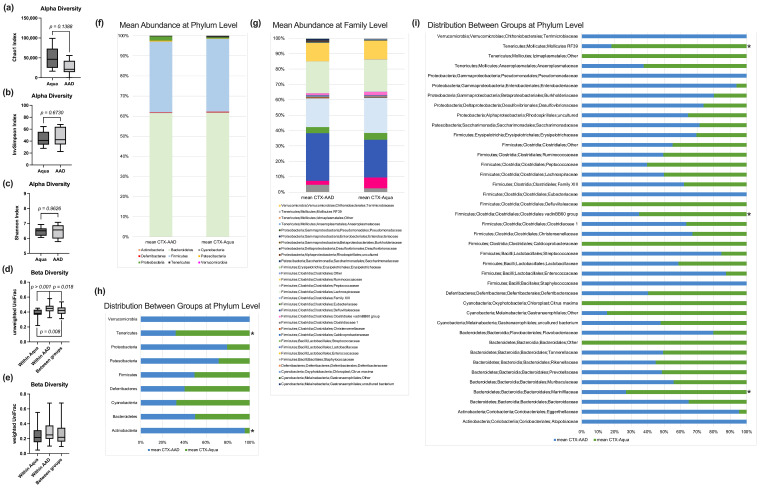
Microbiome analysis. Markers for alpha (**a**–**c**) and beta (**d**,**e**) diversity. Mean relative abundances at the phylum (**f**) and family (**g**) level. Distribution of the mean relative abundances between the groups at the phylum (**h**) and family (**i**) level. * *p* < 0.05.

**Figure 5:**
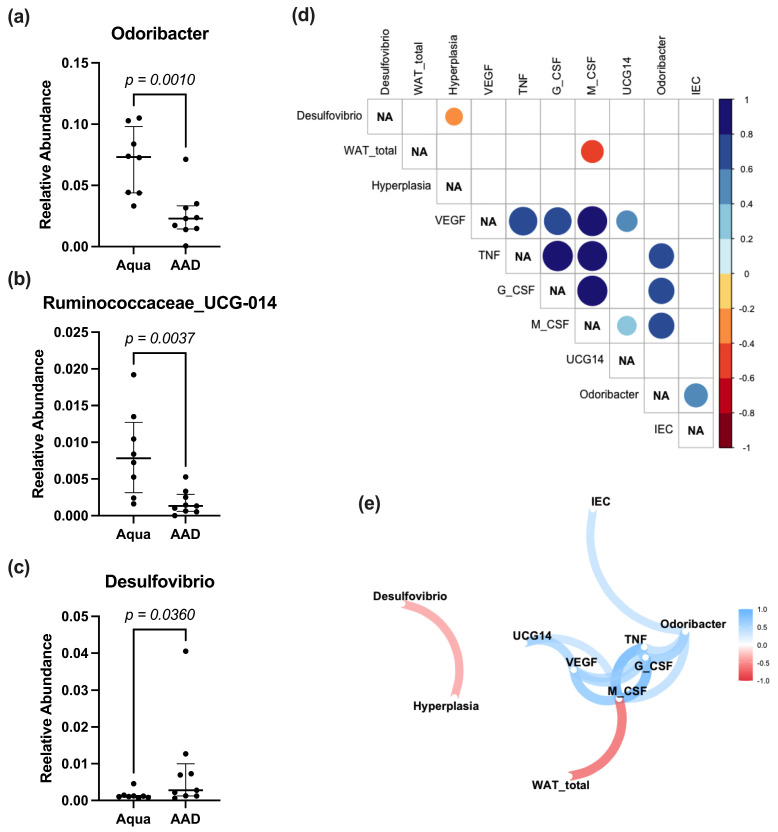
Charts (median and interquartile range) of the relative abundance of species with significant group differences (**a**–**c**); correlation analysis (**d**) (only significant correlations displayed), and correlation path analysis (**e**).

Analysis of the fecal bacterial microbiome revealed no statistically significant differences for the alpha diversity markers Chao1, or the inversed Simpson and Shannon indexes (Figure 4a–c). The unweighted UniFrac measurement showed a significantly smaller distance within the Aqua compared to within the 10 AAD mice, as well as between the groups (Figure 4d), which was not observed in the weighted UniFrac analysis (Figure 4e). At the phylum level, Actinobacteria and Tenericutes were significantly higher in the 10 AAD compared to the Aqua groups (Figure 4h). At the family level, we observed significantly lower *Mollicutes RF39*, *Clostridiales vadinBB60* group and *Marinifilaceae* in the 10 AAD group (Figure 4i). Neither Adonis nor LEfSE analysis yielded significant differences at the species level. However, a direct comparison of the relative abundances at the species level revealed significantly lower levels of *Odoribacter*, *Ruminococcaceae_UCG-014*, with significantly higher levels of *Desulfovibrio* in the 10 AAD group. *Desulfovibrio* negatively correlated with bowel wall hyperplasia, while *Odoribacter* had a significant positive correlation with G-CSF, M-CSF, and TNF-α (Figure 5).

### 3.5. Fecal Volatile Organic Compound Profile

A total of 26 different VOCs could be identified in the headspace of fecal samples; eight of these (Propene, Propanal, 2-Methylbutane, Sevoflurane, Isoflurane, Benzene, n-Propylacetate, and Toluene) could be attributed to room air contamination. The remaining VOCs showed no significant group differences.

## 4. Discussion

In this study, we demonstrate a beneficial effect of the probiotic OMNi-BiOTiC^®^ 10 AAD in a murine model of CTX chemotherapy. Mice treated with the probiotic exhibited reduced systemic inflammation, as well as ameliorated body weight and adipose tissue loss compared to animals in the control group. 

OMNi-BiOTiC^®^ 10 AAD (distributed by Institut AllergoSan, Graz, Austria, and produced by Winclove Probiotics B.V., Amsterdam, The Netherlands) is a commercially available probiotic food supplement composed of nine different probiotic strains: *Lactobacillus acidophilus W55* and *W37*, *Lactobacillus paracasei W20*, *Lactobacillus rhamnosus W71*, *Enterococcus faecium W54*, *Lactobacillus salivarius W24*, *Lactobacillus plantarum W62*, *Bifidobacterium bifidum W23*, and *Bifidobacterium lactis W51*. All these strains belong to species with reported beneficial effects (reviewed in [39]), i.e., preventing constipation, travelers’ diarrhea, antibiotic-associated diarrhea, the prevention and treatment of necrotizing enterocolitis, the reduction of radiation-induced diarrhea, and the reduction of the risk of food allergies [40,41,42,43,44,45]. Hitherto, the main focus of OMNi-BiOTiC^®^ 10 AAD lay in the therapy and prevention of antibiotic-associated diarrhea (AAD) [46,47,48]. Compared to single strain probiotics, synergistic combinations of probiotic bacteria may be beneficial. In this regard, a previous study clearly demonstrated advantages of the multi-species probiotic VSL#3—with a composition (*S. thermophilus*, *E. faecium*, *B. breve*, *B. infantis*, *B. longum*, *L. acidophilus*, *L. plantarum*, *L. casei* and *L. delbrueckii*) close to that of OMNi-BiOTiC^®^ 10 AAD—compared to single and multi-strain probiotics [49].

Although the mechanism of chemotherapy-induced diarrhea and IM is different from that of AAD, VSL#3 has also been examined in these pathologies, showing a decrease in diarrhea induced by irinotecan therapy [50]. Furthermore, VSL#3 treatment was associated with reduced body weight loss, increased crypt proliferation, and an inhibition of epithelial cell apoptosis in the small and large intestine in rats [50]. These findings fueled our hypothesis that OMNi-BiOTiC^®^ 10 AAD may also exhibit a beneficial effect on chemotherapy induced IM.

Intestinal mucositis is one of the most severe adverse effects of anti-tumor chemotherapy, often warranting dose reduction or discontinuation of therapy. During chemotherapy IM develops in different steps involving complex signaling pathways [6]. First, there is a direct DNA injury, the formation of reactive oxygen species, and a release of endogenous molecular pattern molecules from damaged cells in the basal layer of the intestinal epithelial cells, submucosa, and endothelium. This is followed by a release of pro-inflammatory cytokines and apoptosis. In the subsequent course, there is an amplification phase, increasing inflammation and apoptosis, which is followed by ulceration with ablation of the villi, disruption of epithelial cell adhesion, discontinuation of the intestinal barrier, and bacterial translocation.

The mucositis itself, but also the increased levels of pro-inflammatory cytokines, may be responsible for the body weight loss observed in many cases of IM. Body weight loss can be caused by reduced energy uptake, increased energy consumption, or a combination of both. Pain and nausea associated with mucositis, as well as the inflammation itself, may cause anorexia. In this regard, IL-1 exhibits inhibitory effects on the neuropeptide Y mediated appetite regulation [51]. Additionally, pro-inflammatory cytokines (especially IL-1, IL-6, and TNF-α) were shown to be important factors in the development of cachexia, a hypercatabolic state associated with muscle and adipose tissue wasting [51]. Here, we demonstrate reduced chemotherapy-induced body weight loss in animals treated with the multi-species probiotic OMNi-BiOTiC^®^ 10 AAD. This finding confirms those found in many other reports regarding mice [50,52,53] and rats [50,54,55,56]. As the mean calorie consumption was not different between the groups, anorexia does not contribute to body weight loss in this study. Probiotic administered mice exhibited higher WAT weights compared to control animals after CTX treatment, while the weights of other organs were not different, indicating that reduced body weight mainly results from adipose tissue loss in CTX animals. 

Reduced WAT weight over a short period of time is mainly caused by increased degradation of stored lipids. Lipolysis is executed by the two main lipases ATGL and HSL (reviewed in [57,58]). For the activation of ATGL, protein kinase A (PKA) phosphorylates PLIN1, leading to a release of CGI58, with the subsequent activation of ATGL [58]. HSL activity is mainly regulated by the PKA-mediated phosphorylation of HSL itself, as well as of PLIN1. Proinflammatory cytokines such as TNF-α and IL-6 were shown to stimulate lipolysis [59,60,61], linking inflammation and adipose tissue wasting. The in line serum levels of pro-inflammatory cytokines and total WAT of animals significantly correlated with each other. At the mRNA level, we could not find significant differences in the expressions of *Atgl*, *Hsl*, *Plin1*, and *Cgi58*. Similarly, the protein levels of HSL and ATGL did not differ between the groups. The OMNi-BiOTiC^®^ 10 AAD-treated animals, however, showed a trend towards reduced HSL activation, as expressed by the pHSL/tHSL ratio. Furthermore, we encountered a significantly reduced expression of the ATGL-activator CGI58 as a possible mechanism for reduced lipolysis in the 10 AAD animals. Additionally, a trend towards reduced triglyceride hydrolase activity was detected in the WAT of the 10 AAD treated animals, indicating that reduced HSL and CGI58 activated ATGL activity, contributing to the preservation of fat mass in 10 AAD/CTX-treated animals.

In accordance with other studies [16,53,54,56,62,63], gavage feeding of OMNi-BiOTiC^®^ 10 AAD led to a significant reduction in pro-inflammatory cytokine serum levels. As increased levels of pro-inflammatory cytokines play an essential role in the development of IM [6], a reduction in their serum levels by OMNi-BiOTiC^®^ 10 AAD (and other probiotics) is an essential argument for supportive treatment during chemotherapy. Probiotics may directly influence the immune system. VSL#3, for instance, was shown to decrease the expression of toll-like receptor 4, nuclear factor kappa B, and inducible nitric oxide synthetase in ulcerative colitis [19,64]. In patients with pouchitis, VSL#3 administration changed the cytokine profile, as well as the expression of nitric oxide synthase and matrix metalloproteinase [65]. However, systemic inflammation may also be triggered by increased intestinal permeability for pro-inflammatory pathogenic microbe-associated molecular patterns (MAMPs) like lipopolysaccharide, flagellin, or peptidoglycane [66,67]. Cellular loss due to apoptosis, necrosis, or alterations of the inter-epithelial barriers triggered by pro-inflammatory cytokines may cause increased permeation of MAMPs [68]. Probiotics were reported to impact the restoration and stabilization of the intestinal barrier by modulating the expression and distribution of tight junction proteins, increasing the production of the protective mucus layer and the production of short-chain fatty acids (SCFAs) as an energy source for intestinal epithelial cells [6,17,19,69,70,71]. We demonstrated a significant reduction in the intestinal permeability for FITC dextran in animals of the 10 AAD group. We found decreased villus height in the ileum and increased inflammation in the ileum and colon. While there were no significant differences in the ileum, the colon samples showed significantly reduced inflammation and goblet cell loss in the 10 AAD group. Expression analyses of genes associated with intestinal permeability (claudins 2 and 4, occluding 1, tight junction protein 1, myosin light chain kinase, mucin 2, and mucin 3), however, yielded no significant group differences in the ileum or the colon. In contrast to the work of Chang et al. [72], who immunohistologically demonstrated a decrease in apoptotic intestinal cells in a murine FOLFOX model treated with *L. casei variety rhamnosus* (Lcr35), we could not find significant differences in the expression of apoptosis marker genes in the ileum or the colon. While differences in bowel wall inflammation and FITC permeability are possible explanations for the reduced systemic inflammation in the 10 AAD group, the underlying mechanism for this difference remains unclear at present.

In previous studies, we and others demonstrated a significant reduction of *Lactobacillus* and *Bifidobacterium* in the fecal samples of chemotherapy models [12,23]. These deficits described in the literature prompted us to choose OMNi-BiOTiC^®^ 10 AAD (containing different *Lactobacillus* and *Bifidobacterium* strains) as a multispecies probiotic in this study. Despite the amelioration of WAT and body weight loss, as well as decreased inflammation due to OMNi-BiOTiC^®^ 10 AAD supplementation, microbiome analysis revealed no major differences. There were no differences in alpha diversity, and beta diversity analysis showed significant differences in unweighted, but not in weighted UniFrac analysis. Neither ANCOM, nor LEfSE or Random Forest analysis, yielded significant biomarkers for either group. Some significant differences observed in a direct comparison at the phylum, family, and species level are, however, of minor importance. A Spearman correlation analysis linked *Desulfovibrio* to colonic hyperplasia scores, *Ruminococcae-UCG-14* to M-CSF, and VEGF and *Odoribacter* to interepithelial leucocyte counts. Although *Odoribacter* is commonly reported as a beneficial strain [73,74,75], increased levels of *Odoribacter* have been reported in mice with acute colitis, which could be reduced by the administration of VSL#3 [76]. *Ruminococcus* has been reported to be associated with negative health outcomes [77]. Increased levels of *Ruminococcus* have been observed in patients with irritable bowel syndrome and flare-ups of inflammatory bowel disease [78,79]. Furthermore, *Ruminococcus* and *Bacteroidea* were dominant regarding low gene count in obese patients, and they were associated with inflammation and insulin resistance [80]. Similar to *Odoribacter, Ruminococcus* levels were also reduced by the administration of pre- and probiotics, in some studies [81,82]. Since the tests for distinctive biomarkers were negative and only direct comparison at the species level yielded significant differences, the biological effect of the microbial differences we encountered is debatable.

Volatile organic compounds in the headspace of fecal samples are generated during metabolic processes within the intestine and are influenced by the intestinal epithelium, the microbial composition, and diet [83]. In this investigation, we did not find any significant differences in the fecal VOC analysis. This may be due to the lack of major microbial differences between the groups encountered in our microbiome analysis.

### Study Limitations

Regarding lipolysis markers, only six samples per group were available. A higher number of specimens would have been desirable, but lipolysis reduced the amount of available WAT. Although the determination of cecal and/or fecal SCFAs would have been interesting, unfortunately, it was not possible to analyze these biomarkers at the time of the study. Finally, our model was based on cyclophosphamide chemotherapy. It might well be possible that other chemotherapeutical agents or dosage protocols would lead to different results. The different agents and the different animal models used in the literature also make direct comparisons of the results difficult.

## 5. Conclusions

Oral administration of OMNi-BiOTiC^®^ 10 AAD could mitigate cyclophosphamide-induced side effects, such as body weight loss and adipose tissue wasting, as well as bowel wall and systemic inflammation in a murine model. A possible beneficial effect regarding human chemotherapy requires elucidation in future studies.

## Figures and Tables

**Table 1:** Serum cytokine levels of the two groups.

Param.	Unit	Aqua		10 AAD		
		Median	25–75 Perc.	Median	25–75 Perc.	*p*-Value
INF-γ	pg/mL	13.1	3.0–60.3	3.0	3.0–11.2	0.160
IL-1α	pg/mL	18.1	6.3–813.8	1.0	1.0–8.0	**0.022**
IL-1β	pg/mL	7.2	0.4–765.9	0.4	0.4–1.7	**0.021**
IL-6	pg/mL	862.1	263.8–121,076	27.5	27.5–307.9	**0.011**
IL-10	pg/mL	7.9	1.9–236.3	0.1	0.1–1.6	**0.018**
IL-15	pg/mL	8.1	2.6–30.9	0.3	0.3–2.7	**0.018**
IL-17	pg/mL	17.0	1.6–38.6	1.6	1.6–4.7	0.056
MIP-1α	pg/mL	2.2	0.1–167.5	0.1	0.1–0.1	0.076
MIP-1β	pg/mL	1.4	0.9–119.1	0.7	0.6–3.8	**0.042**
MIP-2	pg/mL	99.2	32.9–19,710	26.0	26.0–50.7	**0.024**
MCP-1	pg/mL	552.6	49.6–21,255	51.8	28.3–1161	0.385
TNF-α	pg/mL	59.9	10.9–276.5	1.7	1.7–20.8	**0.024**
G-CSF	pg/mL	3121	1421–4864	510	51.1–1636	**0.021**
GM-CSF	pg/mL	5.1	3.4–48.7	3.4	3.4–3.4	**0.029**
M-CSF	pg/mL	1.4	0.4–3.8	0.3	0.2–0.4	**0.046**
MPO	ng/mL	118.4	60.6–283.9	48.9	29.6–68.7	**0.040**
NE	ng/mL	30.9	6.9–30.9	4.3	2.2–11.6	0.061
LBP	ng/mL	271.1	74.3–2764	125.2	56.5–344.4	0.291
VEGF	ng/mL	16.2	2.2–32.5	0.6	0.1–4.1	**0.047**
TGF-β1	pg/mL	26,143	17,586–31,665	34,677	29,160–44,524	**0.019**
TGF-β2	pg/mL	986.4	696.2–1232	1141	911.6–1330	0.436

Bold: statistically significant values.

## Data Availability

Data are available from the corresponding author upon reasonable request.

## Supplementary Materials

The following supporting information can be downloaded at: https://www.mdpi.com/article/10.3390/nu15163655/s1, Table S1: Primer information.

## References

[1] Davila M., Bresalier R.S. (2008). Gastrointestinal complications of oncologic therapy. Nat. Clin. Pract. Gastroenterol. Hepatol..

[2] Smith C.L., Geier M.S., Yazbeck R., Torres D.M., Butler R.N., Howarth G.S. (2008). *Lactobacillus fermentum* BR11 and fructo-oligosaccharide partially reduce jejunal inflammation in a model of intestinal mucositis in rats. Nutr. Cancer.

[3] Ciorba M.A., Hallemeier C.L., Stenson W.F., Parikh P.J. (2015). Probiotics to prevent gastrointestinal toxicity from cancer therapy: An interpretive review and call to action. Curr. Opin. Support. Palliat. Care.

[4] Sonis S.T. (2004). The pathobiology of mucositis. Nat. Rev. Cancer.

[5] Keefe D.M. (2004). Gastrointestinal mucositis: A new biological model. Support. Care Cancer.

[6] Miknevicius P., Zulpaite R., Leber B., Strupas K., Stiegler P., Schemmer P. (2021). The Impact of Probiotics on Intestinal Mucositis during Chemotherapy for Colorectal Cancer: A Comprehensive Review of Animal Studies. Int. J. Mol. Sci..

[7] Gibson R.J., Keefe D.M., Clarke J.M., Regester G.O., Thompson F.M., Goland G.J., Edwards B.G., Cummins A.G. (2002). The effect of keratinocyte growth factor on tumour growth and small intestinal mucositis after chemotherapy in the rat with breast cancer. Cancer Chemother. Pharmacol..

[8] Gibson R.J., Keefe D.M., Thompson F.M., Clarke J.M., Goland G.J., Cummins A.G. (2002). Effect of interleukin-11 on ameliorating intestinal damage after methotrexate treatment of breast cancer in rats. Dig. Dis. Sci..

[9] Elting L.S., Cooksley C., Chambers M., Cantor S.B., Manzullo E., Rubenstein E.B. (2003). The burdens of cancer therapy. Clinical and economic outcomes of chemotherapy-induced mucositis. Cancer.

[10] Stringer A.M., Gibson R.J., Bowen J.M., Keefe D.M. (2009). Chemotherapy-induced modifications to gastrointestinal microflora: Evidence and implications of change. Curr. Drug Metab..

[11] van Vliet M.J., Harmsen H.J., de Bont E.S., Tissing W.J. (2010). The role of intestinal microbiota in the development and severity of chemotherapy-induced mucositis. PLoS Pathog..

[12] Bron P.A., Kleerebezem M., Brummer R.J., Cani P.D., Mercenier A., MacDonald T.T., Garcia-Rodenas C.L., Wells J.M. (2017). Can probiotics modulate human disease by impacting intestinal barrier function?. Br. J. Nutr..

[13] Yang J., Liu K.X., Qu J.M., Wang X.D. (2013). The changes induced by cyclophosphamide in intestinal barrier and microflora in mice. Eur. J. Pharmacol..

[14] Badgeley A., Anwar H., Modi K., Murphy P., Lakshmikuttyamma A. (2021). Effect of probiotics and gut microbiota on anti-cancer drugs: Mechanistic perspectives. Biochim. Biophys. Acta Rev. Cancer.

[15] Stavropoulou E., Bezirtzoglou E. (2020). Probiotics in Medicine: A Long Debate. Front. Immunol..

[16] Wang Y., Wu Y., Wang Y., Xu H., Mei X., Yu D., Wang Y., Li W. (2017). Antioxidant Properties of Probiotic Bacteria. Nutrients.

[17] Otte J.M., Podolsky D.K. (2004). Functional modulation of enterocytes by gram-positive and gram-negative microorganisms. Am. J. Physiol. Gastrointest. Liver Physiol..

[18] Parada Venegas D., De la Fuente M.K., Landskron G., Gonzalez M.J., Quera R., Dijkstra G., Harmsen H.J.M., Faber K.N., Hermoso M.A. (2019). Short Chain Fatty Acids (SCFAs)-Mediated Gut Epithelial and Immune Regulation and Its Relevance for Inflammatory Bowel Diseases. Front. Immunol..

[19] Toumi R., Abdelouhab K., Rafa H., Soufli I., Raissi-Kerboua D., Djeraba Z., Touil-Boukoffa C. (2013). Beneficial role of the probiotic mixture Ultrabiotique on maintaining the integrity of intestinal mucosal barrier in DSS-induced experimental colitis. Immunopharmacol. Immunotoxicol..

[20] Viaud S., Daillere R., Boneca I.G., Lepage P., Langella P., Chamaillard M., Pittet M.J., Ghiringhelli F., Trinchieri G., Goldszmid R. (2015). Gut microbiome and anticancer immune response: Really hot Sh*t!. Cell Death Differ..

[21] Nami Y., Haghshenas B., Haghshenas M., Abdullah N., Yari Khosroushahi A. (2015). The Prophylactic Effect of Probiotic Enterococcus lactis IW5 against Different Human Cancer Cells. Front. Microbiol..

[22] Sharaf L.K., Sharma M., Chandel D., Shukla G. (2018). Prophylactic intervention of probiotics (L. acidophilus, L. rhamnosus GG) and celecoxib modulate Bax-mediated apoptosis in 1,2-dimethylhydrazine-induced experimental colon carcinogenesis. BMC Cancer.

[23] Castellani C., Singer G., Kaiser M., Obermüller B., Warncke G., Miekisch W., Kolb-Lenz D., Summer G., Pauer T.M., Elhaddad A. (2019). The Effects of Neuroblastoma and Chemotherapy on Metabolism, Fecal Microbiome, Volatile Organic Compounds and Gut Barrier—A Murine Model of Human Neuroblastoma. Pediatr. Res..

[24] Schweiger M., Eichmann T.O., Taschler U., Zimmermann R., Zechner R., Lass A. (2014). Measurement of lipolysis. Methods Enzymol..

[25] Adelman D., Murray J., Wu T., Mäki M., Green P., CP K. (2018). Measuring change in small intestinal histology in patients with celiac disease. Am. J. Gastroenterol..

[26] Erben U., Loddenkemper C., Doerfel K., Spieckermann S., Haller D., Heimesaat M.M., Zeitz M., Siegmund B., Kuhl A.A. (2014). A guide to histomorphological evaluation of intestinal inflammation in mouse models. Int. J. Clin. Exp. Pathol..

[27] Klymiuk I., Bilgilier C., Stadlmann A., Thannesberger J., Kastner M.T., Hogenauer C., Puspok A., Biowski-Frotz S., Schrutka-Kolbl C., Thallinger G.G. (2017). The Human Gastric Microbiome Is Predicated upon Infection with Helicobacter pylori. Front. Microbiol..

[28] Callahan B.J., McMurdie P.J., Rosen M.J., Han A.W., Johnson A.J., Holmes S.P. (2016). DADA2: High-resolution sample inference from Illumina amplicon data. Nat. Methods.

[29] Bolyen E., Rideout J.R., Dillon M.R., Bokulich N.A., Abnet C.C., Al-Ghalith G.A., Alexander H., Alm E.J., Arumugam M., Asnicar F. (2019). Reproducible, interactive, scalable and extensible microbiome data science using QIIME 2. Nat. Biotechnol..

[30] Afgan E., Baker D., Batut B., van den Beek M., Bouvier D., Cech M., Chilton J., Clements D., Coraor N., Gruning B.A. (2018). The Galaxy platform for accessible, reproducible and collaborative biomedical analyses: 2018 update. Nucleic Acids Res..

[31] Quast C., Pruesse E., Yilmaz P., Gerken J., Schweer T., Yarza P., Peplies J., Glockner F.O. (2013). The SILVA ribosomal RNA gene database project: Improved data processing and web-based tools. Nucleic Acids Res..

[32] Bergmann A., Trefz P., Fischer S., Klepik K., Walter G., Steffens M., Ziller M., Schubert J.K., Reinhold P., Kohler H. (2015). In Vivo Volatile Organic Compound Signatures of Mycobacterium avium subsp. paratuberculosis. PLoS ONE.

[33] Miekisch W., Trefz P., Bergmann A., Schubert J.K. (2014). Microextraction techniques in breath biomarker analysis. Bioanalysis.

[34] Kienesberger B., Obermuller B., Singer G., Mittl B., Grabherr R., Mayrhofer S., Heinl S., Stadlbauer V., Horvath A., Miekisch W. (2021). (S)-Reutericyclin: Susceptibility Testing and In Vivo Effect on Murine Fecal Microbiome and Volatile Organic Compounds. Int. J. Mol. Sci..

[35] Pedersen T., Wei T., Simko V. (2021). R Package ‘Corrplot’: Visualization of a Correlation Matrix.

[36] Kuhn M., Jackson S., Cimentada J. (2020). Corrr: Correlations in R.

[37] Wickham H., Averick M., Bryan J., Chang W., D’Agostino McGowan L., François R., Grolemund G., Hayes A., Henry L., Hester J. (2019). Welcome to the {tidyverse}. J. Open Source Softw..

[38] Pedersen T., Nicolae B., Francois R. (2021). Farver: High Performance Colour Space Manipulation.

[39] Fijan S. (2014). Microorganisms with claimed probiotic properties: An overview of recent literature. Int. J. Environ. Res. Public Health.

[40] Chmielewska A., Szajewska H. (2010). Systematic review of randomised controlled trials: Probiotics for functional constipation. World J. Gastroenterol..

[41] Demers M., Dagnault A., Desjardins J. (2014). A randomized double-blind controlled trial: Impact of probiotics on diarrhea in patients treated with pelvic radiation. Clin. Nutr..

[42] Di Gioia D., Aloisio I., Mazzola G., Biavati B. (2014). Bifidobacteria: Their impact on gut microbiota composition and their applications as probiotics in infants. Appl. Microbiol. Biotechnol..

[43] Hempel S., Newberry S.J., Maher A.R., Wang Z., Miles J.N., Shanman R., Johnsen B., Shekelle P.G. (2012). Probiotics for the prevention and treatment of antibiotic-associated diarrhea: A systematic review and meta-analysis. JAMA.

[44] Isolauri E., Rautava S., Salminen S. (2012). Probiotics in the development and treatment of allergic disease. Gastroenterol. Clin. N. Am..

[45] McFarland L.V. (2007). Meta-analysis of probiotics for the prevention of traveler’s diarrhea. Travel. Med. Infect. Dis..

[46] Koning C.J., Jonkers D.M., Stobberingh E.E., Mulder L., Rombouts F.M., Stockbrugger R.W. (2008). The effect of a multispecies probiotic on the intestinal microbiota and bowel movements in healthy volunteers taking the antibiotic amoxycillin. Am. J. Gastroenterol..

[47] Lang F.C. (2010). Use of a multi-species probiotic for the prevention of antibiotic associated diarrhea. Nutra Foods.

[48] van Wietmarschen H.A., Busch M., van Oostveen A., Pot G., Jong M.C. (2020). Probiotics use for antibiotic-associated diarrhea: A pragmatic participatory evaluation in nursing homes. BMC Gastroenterol..

[49] Timmerman H.M., Koning C.J., Mulder L., Rombouts F.M., Beynen A.C. (2004). Monostrain, multistrain and multispecies probiotics—A comparison of functionality and efficacy. Int. J. Food Microbiol..

[50] Bowen J.M., Stringer A.M., Gibson R.J., Yeoh A.S., Hannam S., Keefe D.M. (2007). VSL#3 probiotic treatment reduces chemotherapy-induced diarrhea and weight loss. Cancer Biol. Ther..

[51] Argiles J.M., Busquets S., Garcia-Martinez C., Lopez-Soriano F.J. (2005). Mediators involved in the cancer anorexia-cachexia syndrome: Past, present, and future. Nutrition.

[52] Bastos R.W., Pedroso S.H., Vieira A.T., Moreira L.M., Franca C.S., Cartelle C.T., Arantes R.M., Generoso S.V., Cardoso V.N., Neves M.J. (2016). *Saccharomyces cerevisiae* UFMG A-905 treatment reduces intestinal damage in a murine model of irinotecan-induced mucositis. Benef. Microbes.

[53] Kato S., Hamouda N., Kano Y., Oikawa Y., Tanaka Y., Matsumoto K., Amagase K., Shimakawa M. (2017). Probiotic Bifidobacterium bifidum G9-1 attenuates 5-fluorouracil-induced intestinal mucositis in mice via suppression of dysbiosis-related secondary inflammatory responses. Clin. Exp. Pharmacol. Physiol..

[54] Mi H., Dong Y., Zhang B., Wang H., Peter C.C.K., Gao P., Fu H., Gao Y. (2017). Bifidobacterium Infantis Ameliorates Chemotherapy-Induced Intestinal Mucositis Via Regulating T Cell Immunity in Colorectal Cancer Rats. Cell Physiol. Biochem..

[55] Tang Y., Wu Y., Huang Z., Dong W., Deng Y., Wang F., Li M., Yuan J. (2017). Administration of probiotic mixture DM#1 ameliorated 5-fluorouracil-induced intestinal mucositis and dysbiosis in rats. Nutrition.

[56] Yuan K.T., Yu H.L., Feng W.D., Chong P., Yang T., Xue C.L., Yu M., Shi H.P. (2015). *Bifidobacterium infantis* has a beneficial effect on 5-fluorouracil-induced intestinal mucositis in rats. Benef. Microbes.

[57] Lass A., Zimmermann R., Oberer M., Zechner R. (2011). Lipolysis—A highly regulated multi-enzyme complex mediates the catabolism of cellular fat stores. Prog. Lipid Res..

[58] Nielsen T.S., Jessen N., Jorgensen J.O., Moller N., Lund S. (2014). Dissecting adipose tissue lipolysis: Molecular regulation and implications for metabolic disease. J. Mol. Endocrinol..

[59] Li T., Guo W., Zhou Z. (2021). Adipose Triglyceride Lipase in Hepatic Physiology and Pathophysiology. Biomolecules.

[60] Buhl M., Bosnjak E., Vendelbo M.H., Gjedsted J., Nielsen R.R., K.-Hafstrøm T., Vestergaard E.T., Jessen N., Tonnesen E., Moller A.B. (2013). Direct effects of locally administered lipopolysaccharide on glucose, lipid, and protein metabolism in the placebo-controlled, bilaterally infused human leg. J. Clin. Endocrinol. Metab..

[61] Fruhbeck G., Mendez-Gimenez L., Fernandez-Formoso J.A., Fernandez S., Rodriguez A. (2014). Regulation of adipocyte lipolysis. Nutr. Res. Rev..

[62] Huang L., Chiang Chiau J.S., Cheng M.L., Chan W.T., Jiang C.B., Chang S.W., Yeung C.Y., Lee H.C. (2019). SCID/NOD mice model for 5-FU induced intestinal mucositis: Safety and effects of probiotics as therapy. Pediatr. Neonatol..

[63] Yeung C.Y., Chan W.T., Jiang C.B., Cheng M.L., Liu C.Y., Chang S.W., Chiang Chiau J.S., Lee H.C. (2015). Correction: Amelioration of Chemotherapy-Induced Intestinal Mucositis by Orally Administered Probiotics in a Mouse Model. PLoS ONE.

[64] Bibiloni R., Fedorak R.N., Tannock G.W., Madsen K.L., Gionchetti P., Campieri M., De Simone C., Sartor R.B. (2005). VSL#3 probiotic-mixture induces remission in patients with active ulcerative colitis. Am. J. Gastroenterol..

[65] Shen J., Zuo Z.X., Mao A.P. (2014). Effect of probiotics on inducing remission and maintaining therapy in ulcerative colitis, Crohn’s disease, and pouchitis: Meta-analysis of randomized controlled trials. Inflamm. Bowel Dis..

[66] Al-Sadi R., Boivin M., Ma T. (2009). Mechanism of cytokine modulation of epithelial tight junction barrier. Front. Biosci..

[67] Carvalho F.A., Koren O., Goodrich J.K., Johansson M.E., Nalbantoglu I., Aitken J.D., Su Y., Chassaing B., Walters W.A., Gonzalez A. (2012). Transient inability to manage proteobacteria promotes chronic gut inflammation in TLR5-deficient mice. Cell Host Microbe.

[68] Boyd J.H., Divangahi M., Yahiaoui L., Gvozdic D., Qureshi S., Petrof B.J. (2006). Toll-like receptors differentially regulate CC and CXC chemokines in skeletal muscle via NF-kappaB and calcineurin. Infect. Immun..

[69] Dai C., Zhao D.H., Jiang M. (2012). VSL#3 probiotics regulate the intestinal epithelial barrier in vivo and in vitro via the p38 and ERK signaling pathways. Int. J. Mol. Med..

[70] Mennigen R., Nolte K., Rijcken E., Utech M., Loeffler B., Senninger N., Bruewer M. (2009). Probiotic mixture VSL#3 protects the epithelial barrier by maintaining tight junction protein expression and preventing apoptosis in a murine model of colitis. Am. J. Physiol. Gastrointest. Liver Physiol..

[71] Touchefeu Y., Montassier E., Nieman K., Gastinne T., Potel G., Bruley des Varannes S., Le Vacon F., de La Cochetiere M.F. (2014). Systematic review: The role of the gut microbiota in chemotherapy- or radiation-induced gastrointestinal mucositis—Current evidence and potential clinical applications. Aliment. Pharmacol. Ther..

[72] Chang C.W., Liu C.Y., Lee H.C., Huang Y.H., Li L.H., Chiau J.C., Wang T.E., Chu C.H., Shih S.C., Tsai T.H. (2018). Lactobacillus casei Variety rhamnosus Probiotic Preventively Attenuates 5-Fluorouracil/Oxaliplatin-Induced Intestinal Injury in a Syngeneic Colorectal Cancer Model. Front. Microbiol..

[73] Yue Y., He Z., Zhou Y., Ross R.P., Stanton C., Zhao J., Zhang H., Yang B., Chen W. (2020). *Lactobacillus plantarum* relieves diarrhea caused by enterotoxin-producing *Escherichia coli* through inflammation modulation and gut microbiota regulation. Food Funct..

[74] Zhou J., Li M., Chen Q., Li X., Chen L., Dong Z., Zhu W., Yang Y., Liu Z., Chen Q. (2022). Programmable probiotics modulate inflammation and gut microbiota for inflammatory bowel disease treatment after effective oral delivery. Nat. Commun..

[75] Vincent C., Mehrotra S., Loo V.G., Dewar K., Manges A.R. (2015). Excretion of Host DNA in Feces Is Associated with Risk of Clostridium difficile Infection. J. Immunol. Res..

[76] Meng X.C., Wang Y.N., Yan P.G., Li Y.H., Wang H.Y., Qian J.M., Li J.N. (2019). Effect of VSL#3 and S.Boulardii on intestinal microbiota in mice with acute colitis. Zhonghua Yi Xue Za Zhi.

[77] Hills R.D., Pontefract B.A., Mishcon H.R., Black C.A., Sutton S.C., Theberge C.R. (2019). Gut Microbiome: Profound Implications for Diet and Disease. Nutrients.

[78] Hall A.B., Yassour M., Sauk J., Garner A., Jiang X., Arthur T., Lagoudas G.K., Vatanen T., Fornelos N., Wilson R. (2017). A novel *Ruminococcus gnavus* clade enriched in inflammatory bowel disease patients. Genome Med..

[79] Rajilic-Stojanovic M., Jonkers D.M., Salonen A., Hanevik K., Raes J., Jalanka J., de Vos W.M., Manichanh C., Golic N., Enck P. (2015). Intestinal microbiota and diet in IBS: Causes, consequences, or epiphenomena?. Am. J. Gastroenterol..

[80] Cotillard A., Kennedy S.P., Kong L.C., Prifti E., Pons N., Le Chatelier E., Almeida M., Quinquis B., Levenez F., Galleron N. (2013). Dietary intervention impact on gut microbial gene richness. Nature.

[81] Rios J.L., Bomhof M.R., Reimer R.A., Hart D.A., Collins K.H., Herzog W. (2019). Protective effect of prebiotic and exercise intervention on knee health in a rat model of diet-induced obesity. Sci. Rep..

[82] Valles-Colomer M., Falony G., Darzi Y., Tigchelaar E.F., Wang J., Tito R.Y., Schiweck C., Kurilshikov A., Joossens M., Wijmenga C. (2019). The neuroactive potential of the human gut microbiota in quality of life and depression. Nat. Microbiol..

[83] Ahmed I., Greenwood R., Costello Bde L., Ratcliffe N.M., Probert C.S. (2013). An investigation of fecal volatile organic metabolites in irritable bowel syndrome. PLoS ONE.

