# Supplementary material for: Probiotic OMNi-BiOTiC® 10 AAD Reduces Cyclophosphamide-Induced Inflammation and Adipose Tissue Wasting in Mice
Source: Nutrients. 2023 Aug 20;15(16):3655. doi: 10.3390/nu15163655 (PMC10458463; doi:10.3390/nu15163655)
Supplement: Supplementary file 1 [file nutrients-15-03655-s001.zip › nutrients-2520349-supplementary.pdf]

Supplementary Data

Table S1: Primer information. For the primer design genes were identified from the National Center for Biotechnology Information database (www.ncbi.nlm.nih.gov/gene/). All primers were designed with the Primer designing tool Primer-BLAST (www.ncbi.nlm.nih.gov/tools/primer-blast/index.cgi?LINMK_LOC=BlastHome) using the preset standard settings. Ms…mouse; Seq…Sequence; RefSeq…Accession number; FW…forward; RV…reverse.

msACTB	**Gene ID: 11461**	
**Seq**	FW: TCAGCAAGCAGGAGTACGATG	RV: AACGCAGCTCAGTAACAGTCC
**RefSeq**	NM_007393.5	
msNFKB	**Gene ID: 18033**	
**Seq**	FW: GTGGAGGCATGTTCGGTAGT	RV: CACCGTAGGGAGGAAATCCG
**RefSeq**	NM_008689.2	
msHMBS	**Gene ID: 15288**	
**Seq**	FW: GCGGAGTCATGTCCGGTAA	RV: ATCACCCTCATCTTTGAGCCG
**RefSeq**	NM_013551.2	
msTJP1	**Gene ID: 21872**	
**Seq**	FW: GAGCCCTCCGATCATTCCAC	RV: CCCCAGGTTTAGACATTCGCT
**RefSeq**	NM_009386.2	
msOCCLUDIN-1	**Gene ID: 18260**	
**Seq**	FW: CTTATCTTGGGAGCCTGGACAT	RV: CTTTCAAAAGGCCTCACGGAC
**RefSeq**	NM_008756.2	
msCLAUDIN 4	**Gene ID: 12740**	
**Seq**	FW: CCATGGAACCCTTCCGTTGA	RV: ACCCGTCCATCCACTCTACA
**RefSeq**	NM_009903.2	
msCLAUDIN 2	**Gene ID: 12738**	
**Seq**	FW: ACGTCCAGTGCAATGTCCTC	RV: GCCACTCTGTCCTTAGCTCG
**RefSeq**	NM_016675.4	
msMLCK	**Gene ID: 107589**	
**Seq**	FW: ACAGCCAAGTTCGAAGGGAG	RV: GGTGATGGCTTGCCCTTTTC
**RefSeq**	NM_139300.3	
msMUCIN2	**Gene ID: 17831**	
**Seq**	FW: GACCTGACAATGTGCCCAGA	RV: ACAATGCCACTTCCACCCTC
**RefSeq**	NM_023566.4	
msMUCIN3	**Gene ID: 666339**	
**Seq**	FW: ACTGGTGGAGAGCGTAGAGA	RV: CGGTCCTGTAGCTTCTCACTG
**RefSeq**	NM_010843.2	
msBAX	**Gene ID: 12028**	
**Seq**	FW: CGTGGTTGCCCTCTTCTACTT	RV: ATGGTTCTGATCAGCTCGGG
**RefSeq**	NM_007527.3	
msBAD	**Gene ID: 12015**	
**Seq**	FW: AGCAACATTCATCAGCAGGGA	RV: TACGAACTGTGGCGACTCC
**RefSeq**	NM_007522.3	
msCASPASE3	**Gene ID: 12367**	

**Seq**	FW: CAGTGTCCTGCGGCGG	RV: ATCCACTGAGGTTTTGTTGTTCTC
**RefSeq**	NM_009810.3	
**msLAMIN B1**	**Gene ID: 16906**	
**Seq**	FW: TGAGGCTGTACAAGGAAGAGC	RV: ATCTCTGAGGAGAGTCTGGCA
**RefSeq**	NM_010721.2	
**msBAK 1**	**Gene ID: 12018**	
**Seq**	FW: CCTTCGGGGTCTTCGTCTTT	RV: GGAGACCACCGTCACTTGTC
**RefSeq:**	NM_007523.3	
**msBCL2**	**Gene ID: 12043**	
**Seq**	FW: GACTGAGTACCTGAACCGGC	RV: GCATGCTGGGGCCATATAGT
**RefSeq**	NM_009741.5	
**msATGL**	**Gene ID: 66853**	
**Seq**	FW: GCCAACGCCACTCACATCTA	RV: TAATGTTGGCACCTGCTTCAC
**RefSeq**	NM_025802.3	
**msHSL**	**Gene ID:**	
**Seq**	FW: AGAAGGATCGAAGAACCGCA	RV: CGACTGTGTCATCGTGCGTA
**RefSeq**	NM_010719.5	
**msPLIN1**	**Gene ID: 103968**	
**Seq**	FW: CCACCTGGAGGAAAAGATCCC	RV: GATGGTGCCCTTCAGTTCAGA
**RefSeq**	NM_001113471.1	
**msCGI58**	**Gene ID: 67469**	
**Seq**	FW: GGTGTCCCACATCTACATCACA	RV: CCATTGGATATGCGCACAGG
**RefSeq**	NM_026179.2	
